# Exploring the experiences of school-going children with HIV in Eswatini: A qualitative inquiry

**DOI:** 10.4102/phcfm.v16i1.4472

**Published:** 2024-06-29

**Authors:** Nomathemba Nxumalo, Zelda Janse van Rensburg, Wanda Jacobs

**Affiliations:** 1 Department of Nursing, Faculty of Health Sciences, University of Johannesburg, Johannesburg, South Africa; 2 Department of Nursing, Faculty of Health Sciences, University of Eswatini, Mbabane, South Africa

**Keywords:** children, Eswatini, human immunodeficiency virus, qualitative research, school

## Abstract

**Background:**

Infection by human immunodeficiency virus (HIV) is a major disease in children, affecting an estimated 1.8 million children and adolescents worldwide. Eswatini has the highest prevalence of HIV in the world. Only 76% of children in Eswatini are on anti-retroviral treatment.

**Aim:**

This study aimed to gain an in-depth understanding of the lived experience of school-going children with HIV in Eswatini. Being aware of these children’s experiences can assist schools in supporting them.

**Setting:**

The study was conducted in four primary health care facilities in Eswatini.

**Methods:**

Employing a qualitative, exploratory, descriptive research design, 12 school-going children with HIV were interviewed through semi-structured face-to-face interviews. The data were coded, categorised and clustered into themes and sub-themes using Georgi’s data analysis. Ethical considerations and measures to ensure trustworthiness were adhered to throughout the study.

**Results:**

The findings revealed three themes: Experiences after HIV disclosure, experience of disclosure and discrimination, and experience of desire to fulfil educational needs. Six sub-themes were identified: A feeling of sadness and worry relating to knowledge of HIV diagnosis, a desire to disclose their status to their teachers but not to their peers, a need for protection against discrimination, a desire to learn, illness affecting their learning and expectation for teachers to be supportive in their educational needs.

**Conclusion and contribution:**

The findings of the study guided recommendations that may assist, the Eswatini Ministry of Health, schools, parents and caregivers, and siblings to support school-going children with HIV.

## Introduction

Human immunodeficiency virus (HIV) infection is a major disease in children, affecting an estimated 1.8 million children and adolescents worldwide.^1^ The total number of children born with HIV has been decreasing over the decades because of the success of preventative measures such as prevention of mother-to-child transmission of HIV.^2^ Despite this recent success in HIV transmission reduction, children who were infected during the prenatal period are now school-aged, with a significant proportion in adolescence and early adulthood.^3^ According to the Joint United Nations Programme on HIV (UNAIDS) and Acquired Immunodeficiency Syndrome (AIDS), 84 000 HIV and AIDS-related deaths were recorded globally, with most of these deaths occurring in sub-Saharan Africa among children aged 0–14 years.^4^

The provision of anti-retroviral treatment (ART) has reduced the number of AIDS-related deaths globally by 52%.^4^ Anti-retroviral treatment is especially important in reducing the mortality from HIV in children aged 10 years and younger.^1^ The aim of ART is to suppress viral replication and recover and preserve the immune system^1^ and is therefore vital for children infected with HIV to adhere to treatment. Access to ART has been expanded in all countries, especially in sub-Saharan Africa.^4^ The availability of ART drastically reduces the morbidity and mortality of children infected with HIV and makes the disease a chronic disease rather than a fatal one.^1^

Because of the stigmatised nature of HIV, disclosure of a child’s disease is often challenging.^5^ Many aspects can negatively affect children who discover they are HIV positive. For instance, these children are likely to develop mental health problems, especially if their parents also experience chronic health problems.^6^ Children who discover they are HIV positive and must take ART often struggle with whether they should share their diagnosis with their peers or keep it private. This is particularly difficult for younger children who may not even understand what being HIV positive means.^7^ Their internal struggle may become even more challenging when these children reach adolescence and have not dealt with their deep psychological issues relating to their HIV-positive status. Human immunodeficiency virus-positive children also face ongoing internal challenges in forming peer relationships.^7^ This internal battle may impact everyday behaviours as school-going children try to fit in among their peers. Particular attention needs to be paid to children before they reach adolescence, where they typically face more of life’s complexities and decisions.^8^

Eswatini, a country in the eastern flank of Southern Africa and within sub-Saharan Africa, has the highest prevalence of HIV in the world.^9^ About 11 000 children (0–14 years) had HIV in Eswatini as of 2018.^9^ Only 76% of these children were on ART. Approximately 45 000 children have also been orphaned because of AIDS-related illnesses. The number of new infections and AIDS-related deaths has reduced to fewer than 1000 each year.^10^ In 2021, according to the UNAIDS, 27.3% of people aged 15–49 years lived with HIV in Eswatini, and HIV/AIDS is still the number one cause of death in this country.^9^

Health programmes are consistently implemented in Eswatini, aiming to reduce some of the physical, emotional and psychological pressures on children with HIV. A community-based programme called Bantwana Schools Integrated Programme (BSIP) in the Lubombo region of Eswatini supports school committees and schools to provide a wide range of comprehensive services for vulnerable children such as integrated socio-economic services, health and HIV interventions, social and child protection, as well as educational interventions.^11^ The Bantwana programme further has an ‘Insika Ya Kusasa’ project that has reached six constituencies in Eswatini.^11^ The project is covering 19 clubs of Children and Adolescents Living with HIV (C and ALHIV). The project implements programmes to ensure HIV prevention for young people as well as care and treatment that support the country’s 95:95:95 goals.^11^ The project also aims at mobilising and distributing HIV self-testing among young women and increasing HIV case identification and linkages to care.^11^ Furthermore, the Ministry of Education in Eswatini also launched the Inqaba Schools Programme which is a school management guide that focuses on a rights-based approach to provide an inclusive school-based integrated model of care and support to orphans and other vulnerable children in Eswatini.^12^ This model supports the notion of the school being a safe sanctuary for children who are affected and infected by HIV. The model embraces the idea that a school is an ‘Inqaba’ (siSwati word for fortress) and not just a place for academic gain but is open to the community and the family to build a support structure for the ultimate welfare of the child.^12^ These programmes are, however, not focused on the needs of school-going children with HIV, and therefore, school-going children with HIV in Eswatini still lack support and require focused interventions informed by empirical research.

Human immunodeficiency virus’s effect on children’s education includes an overt decline in school enrolment, attendance and progress. Paying closer attention to the school-going HIV-infected pre-adolescent group could help ensure that these children are initiated and retained in care, resulting in positive clinical outcomes. Support measures from all aspects of their life, including their school, could prevent them from facing challenges during adolescence, such as treatment fatigue, loss to follow-up and the intense social pressure to sexually experiment to ‘fit in’. If children are not adequately supported, their school trajectory may be affected by illness, frequent absenteeism, stigmatisation, humiliation and exclusion.^13^

The burden of HIV on these children has been researched widely, especially in relation to the role of the family, health care providers and the health care workers^14,15,16,17,18,19^; however, not much has been reported on the experience of the school-going child with HIV, particularly in a low socio-economic environment with a high prevalence of HIV such as Eswatini. In the area of exploring the schooling experience of the child with HIV, there is a need to understand how these children experience living with HIV, accessing and adhering to ART and treatment of ailments.

The research from which the article is drawn originated from a larger doctoral (PhD) study that explored the experiences of school-going children living with HIV, their caregivers/parents and siblings. In this article, we describe the experiences of school-going children with HIV in Eswatini. Being aware of these children’s experiences can assist schools to support these children. The data also enabled the researcher, in the larger study (PhD study), to develop a continuum of support for school-going children with HIV in Eswatini, with a focus on the children, parent and caregivers, siblings and teachers.

## Research designs and methods

### Study design and setting

A qualitative, descriptive study was conducted to explore and describe the experiences of school-going children with HIV in Eswatini. Children aged 8–13 years with HIV were purposefully selected from four health facilities in the Hhohho region where they access ART and interviewed through individual semi-structured in-person interviews. The researcher chose the age group of 8–13 years as they were in primary school and likely to undergo a myriad of experiences related to their HIV-positive status. Given the participant’s age, they could clearly articulate their experiences.

The Kingdom of Eswatini, previously known as Swaziland, is a landlocked country, surrounded by the eastern flank of South Africa and Mozambique located in the sub-Saharan Africa with a population of about 1.1 million people.^20,21^ The majority of the Eswatini population (63%) lives below the poverty line.^22^ The HIV prevalence in Eswatini is 27.3% among those aged 15 years and older, which is the highest percentage in the world.^23,24^ The current HIV prevalence is at 2.8% among those aged 0–14 years,^25^ with AIDS-related illness being the lead cause of death for under-fives.^25^ The Hhohho region is in the northern part of Eswatini and is characterised by the second highest HIV prevalence (27.5%) in the country after Manzini (31.9%)^25^ that includes children.

The four health facilities included one referral hospital with several specialised departments offering curative services and chronic disease management as well as admissions. The hospital also has an HIV clinic within the premises which caters for HIV management of children as well as adults. In the hospital, Tuesdays are dedicated to clinic days for children with HIV. The children with HIV are, however, allowed to come on any other day, including a Tuesday, to consult for minor ailments. The other three facilities included primary health care clinics in the peri-urban and rural areas of the Hhohho region. Children access HIV management services daily in these facilities if they are scheduled. However, to allow the children who have transitioned into teenage hood to also have their support group, they use their Teen Club (where ALHIV are assisted to build positive relationships, improve their self-esteem and acquire life skills through peer mentorship). In all the facilities, the children often visit the clinics with their parents and caregivers who are also treatment supporters.

### Study population and sampling strategy

Twelve participants were purposively recruited to participate in the study. The inclusion criteria were children aged 8–13 years who have been on ART for more than 6 months and attended one of the four selected health facilities to access their ART. The children needed to live at home and attend school daily. During recruitment, a total of 24 children were approached, and the study was explained to them. However, only 12 of these children were enrolled in the study. Some refused participation for reasons such as non-disclosure, not being willing to discuss their experience or not being accompanied by their parent and caregiver to the clinic on the specific day. The number of school-going children sampled from each facility was not the same because of the aforementioned reasons. No specifications to gender were made in the inclusion criteria.

Firstly, the study was explained to the health facility managers to obtain permission to access the parents and caregivers and children at the clinic. Secondly, once permission was obtained from the health facility managers, the parents and caregivers and children were approached during their visit to the HIV/ART clinic, and the study was explained to the parents and caregivers and the children. The children were requested to avail themselves for possible participation in the study once their parents and caregivers gave written consent for them to participate in the study as they were minors (below 18 years old). The children thereafter signed ascent to participate in the study. Only those children who ascent to participate despite the parents’ consent were included in the research. Data were collected at the health facility or at another setting preferred by the child’s parent and caregiver such as their home. Most of the participants’ parents and caregivers preferred that the data should be collected at the health facility rather than at their home. A few of the participants were interviewed at home. The interviews were conducted between September and March 2020.

### Data collection and instrumentation

Data were collected through in-depth, semi-structured, individual in-person interviews. One open-ended question was asked: ‘Can you tell me what it is like for you to have HIV and go to school?’ The researcher used communication skills and interview techniques, like probing, clarification, silence, paraphrasing and active listening to elicit further dialogue.^26^ This allowed the participants to narrate their experiences freely in their own words after establishing rapport. One interview was done as a pilot interview. This was done to determine the central question’s effectiveness in obtaining the required data and answering the research question. After the pilot interview, it was determined that the research question was effective, and data were viewed as valuable and were therefore included in the data set. All interviews were recorded using an electronic audio-recorder to preserve each participant’s response with permission from the parents, caregivers and the participants. The interviews were conducted in the native language, siSwati, for the participants to easily express themselves. Each interview session lasted approximately 45 min. Field notes and observational notes were taken by the researcher after the interviews. By the 12th concluded interview, no new themes and categories emerged. The independent coder reviewed the data and agreed that data saturation was reached. The interviews were conducted in three phases, namely, the introduction phase, working phase and termination phase. During the introduction phase, the participant was probed by asking questions such as^26^ ‘tell me more …’ to give more useful information. During the working phase, the participant’s experiences were explored by clarification, active listening and taking observational notes.^26^ Questions such as ‘when you say uncomfortable, what do you mean …’ were used. During the termination phase, the researcher ended the interview using a closing question: ‘do you have anything else to add?’.^26^

### Data analysis

Georgi’s phenomenological data analysis steps were followed for the analysis of data to code, categorise and cluster the data into themes and categories.^27^ The thematic analysis was done manually according to Giorgi included first assuming a phenomenological attitude, then reading the entire written account for a sense of the whole, thereafter delineating meaning units, followed by transforming the meaning units into psychologically sensitive statements of lived meanings and lastly synthesising a general psychological structure of the experience based on the constituents of the experience. All the audio-recorded interviews were transcribed verbatim manually by the researcher and translated into English and translated back into siSwati to maintain content preservation, whereafter they were analysed. The data were manually analysed by the researcher and an independent coder, experienced in qualitative data analysis. The researcher’s immersion in the data was achieved by prolonged engagement in reading the data repeatedly and analysing the verbatim transcripts manually.^27^ A consensus discussion was held between the researcher, independent coder and supervisors to finalise the themes and categories. Three themes emerged, and an inductive process was used to derive categories from the main themes.^28^ Field notes of body language cues, nuances and facial expressions assisted in supporting the categories.

### Ensuring trustworthiness

During the interviews, the researcher paraphrased, clarified and summarised to gain a clear understanding of the participants’ voices. Credibility was ensured by asking one carefully designed central research question using interview techniques like probing questions to explore the participants’ lived experience, engage with the participants during the interview and conduct member check post-interview.^28^ Although the central question was in English, the researcher is fluent in siSwati and could relate summaries, probing and clarifying responses with ease when the participant could not understand the English. Phenomenological reduction and bracketing were adhered to by the researcher by keeping a personal reflective journal to bracket her feelings and opinions throughout the study.^28^ Some of the feelings recorded in the reflected journal were feelings of sadness from seeing how some of the children have suffered and still do from the impact of HIV on their lives. It was also noted how amazing it was that some of the children displayed resilience in being HIV positive. From the start of the interviews, the researcher also reflected and wrote down her own experience related to family members with HIV, ensuring that any personal feelings that arise during the data collection and analysis process did not cloud the analysis and interpretation of data.^28,29^ A rich description of the research methods, study setting and sampling approach and participant’s demographic characteristics contributed to the transferability of the findings.

### Ethical considerations

Permission to conduct the study was obtained from the Research Ethics Committee (approval number REC-01-157-2018) and the Higher Degrees Committee (approval number HDC-01-109-2018) at a University in Gauteng, South Africa on 16 January 2019. Permission to conduct the study in Eswatini was granted by the Eswatini National Health Research Review Board (approval number SHR092/2019). The following ethical principles were adhered to: respect for autonomy, beneficence, non-maleficence and justice. The participants received information letters that stated the study’s aims, objectives and potential risks. The participants also exercised their self-determination and autonomy. Parents and caregivers gave written consent for the child to participate in the study. The children gave written assent to participate in the study and were not forced to participate if they did not agree, even if the parents and caregivers gave consent. Once the interviews commenced, the participants were assured that their participation was voluntary and that they had a right to withdraw at any time during the interview without any negative consequences. During the consent and ascent for the study, potential participants were informed that they could withdraw at any stage during the research and were provided with the contact details of the researcher. To date, none of the participants indicated their wish to withdraw from the research. Should the child have experienced any psychological discomfort before, during or after the interviews, they were asked to inform the researcher immediately and would have been referred for appropriate psychological counselling. No psychological discomfort was reported before, after or during the interviews. Confidentiality was ensured by providing the participants with participant numbers (such as C1) to protect the real identity of the participants. Interviews conducted at the health facilities were conducted in a private room. The researcher, supervisors and independent coder were the only ones with access to the audio recordings and the transcriptions. The audio recordings and the transcriptions were kept in a password-protected file on a password-protected laptop of the researcher. The password was only known by the researcher. The consent forms were scanned and electronically stored in a separate password-protected file to prevent bridging the devices. The audio recordings and the transcriptions will be destroyed 2 years after the publication of the data as per University Institutional Policy.

## Results

### Demographic data of the participants

The demographic data of the participants are presented in Table 1. The participant’s demographic information included the participant’s gender, age, school grade, period of HIV disclosure and the primary caregiver.

**TABLE 1:** Demographic data of the participants.

Participant number	Age (years)	School grade	Period of HIV disclosure	Primary caregiver
C1	12	Grade 6	3 years	Mother
C2	9	Grade 4	2 years	Grandfather
C3	11	Grade 3	2 years	Grandfather
C4	12	Grade 4	5 years	Mother
C5	11	Grade 6	2 years	Mother
C6	14	Grade 5	2 years	Mother
C7	11	Grade 5	8 years	Mother
C8	13	Grade 6	3 years	Aunt
C9	12	Grade 4	1 year	Mother
C10	8	Grade 4	3 years	Mother
C11	12	Grade 5	3 years	Aunt
C12	9	Grade 2	1 year	Mother

HIV, human immunodeficiency virus.

All the participants were between the ages of 8 and 13 years. School grade levels ranged from Grade 2 to Grade 6. The majority have had their HIV status disclosed to them for 1–3 years while one child has known their HIV status for 8 years. The primary caregiver for most of the participants was their mother, while for some, it was their grandfather or aunt with whom they are living.

### Themes and sub-themes

Three themes are discussed in this article, namely, experiences after HIV disclosure, experience of disclosure and discrimination, and the experience of desire to fulfil educational needs. Table 2 shows the emerging themes and sub-themes discussed in this article.

**TABLE 2:** Emerging themes and sub-themes.

Themes	Sub-themes
Experiences after HIV disclosure.	A feeling of sadness and worry relating to knowledge of HIV diagnosis.
Experience of disclosure and discrimination.	A desire to disclose their status to their teachers but not to their peers. A need for protection against discrimination.
Experience of desire to fulfileducational needs.	A desire to learn. Illness affecting their learning. An expectation for teachers to be supportive in their educational needs.

HIV, human immunodeficiency virus.

### Theme 1: Experiences after human immunodeficiency virus disclosure

The feelings the participants experienced after their HIV status were disclosed to them, including feelings of sadness, concern and feelings towards various health challenges such as continuous coughing, rashes, fatigue, ear problems, stomach problems, etcetera. The participants also struggled to understand the disease.

#### Sub-theme 1: A feeling of sadness and worry relating to knowledge of human immunodeficiency virus diagnosis

There was an emphasis on a feeling of worry after the children were informed about their HIV status and trying to cope without fully understanding what it really means to be HIV positive. The participants felt mostly sad after their HIV status was disclosed to them. Some of the participants did not fully understand what the diagnosis really meant for them in the long term. It was evident that living with HIV led them on a rollercoaster of emotions; sometimes, they would feel happy even though they were HIV positive, but other times, they felt sad because of the diagnosis. The participants mentioned:

‘It’s not a pleasant experience to live with HIV’. (C1)

‘It happens that I sometimes feel bad …’. (C3)

‘It feels OK, sometimes good and sometimes bad’. (C8)

The participants also mentioned that they often felt different from their peers. One participant mentioned:

‘I feel alone and different from others, but they explained to me that I’m not the only one living with the virus, there are many others’. (C7)

The reality of the need for long-term treatment created exhaustion among the children, especially as they had been on treatment since they were very young, even before they were disclosed to. One participant shared:

‘Sometimes I feel tired of taking the pills’. (C3)

### Theme 2: Experience of disclosure and lack of trust

The participants wished to disclose their diagnosis to their teachers, but their parents and caregivers had strong resistance against disclosing because of a lack of trust in the teachers. The participants were, however, not in favour of disclosing their status to their peers for the fear of discrimination.

#### Sub-theme 1: A desire to disclose their status to their teachers but not to their peers

The participants wanted to disclose their HIV status to their teachers mostly because it made it easier for them to request permission to go to the hospital when they needed to. The participants also mentioned that they wanted their teachers to know their HIV status to avoid awkward questions when they were late arriving at school on clinic days. The participants mentioned:

‘I wish … to tell [*my*] teacher that I am taking pills because when I am late on Tuesdays, she [*teacher*] says “you woke up late” … yet I am from hospital, I tell her I was collecting pills …’. (C3)

‘The teachers tend to ask me a lot of questions whenever I go to the hospital and this to me … is uncomfortable’. (C4)

‘When a child, let me make an example, has to go to the clinic to get pills, when he ask for a pass out note they [*teachers*] shouldn’t say he is skipping school. It will happen that the child tells them that he is going to get pills from the clinic. They should not keep on asking what the pills are for’. (C6)

The participants also mentioned that if the teachers know their HIV status, the teacher, parents and caregivers can better assist them. One participant mentioned:

‘They [*teachers and parents*] can work together by ensuring that I get to hospital whenever I need to … And ensuring that there’s no problem when I come back to school from the hospital’. (C8)

The participants also felt that if they can disclose their HIV status to the teachers, the teacher can be a form of support to them in dealing with their disease or learning. Two participants mentioned:

‘I feel bad because it [*not being free to talk to teachers*] prevents me from [*venting*] my concerns or problems because at times there are things that bother us at school’. (C7)

‘I should also be free to communicate about matters that concern my well-being or things that bother me as a pupil. For example, I should be able to tell the teacher about my problems or things that make me uncomfortable in class. Parents and teachers should make this easier to do because sometimes you find that there are things that bother me and need me to report them to someone, so I am comfortable’. (C7)

The participants mentioned that not disclosing their status to their teachers creates a sense that they are not taking their learning seriously. When asked why they are late for school or why they had to go to the clinic or hospital, it often creates an uncomfortable feeling and makes them unsure of how to respond. One participant mentioned:

‘The teacher normally asks what I had gone to do at the hospital then I don’t know how to respond to this question’. (C4)

The participants stated that although they were keen to disclose their HIV status to their teachers, they did not feel the same way in disclosing their status to their peers. The participants verbalised a strong fear of discrimination by their peers. Some participants mentioned:

‘They do discuss [*the peers*] and then they look at you. It hasn’t happened to me. There is this other child who takes pills whom they discuss, and they would sometimes look at her. It was better because they didn’t know my status. Only my teacher knew, and she didn’t discuss (*sic*) me’ (C11)

‘It made me feel bad because my friends kept asking me questions, and I won’t tell them’. (C4)

The participants mentioned that although they did not disclose their HIV status to their peers, the teachers to whom they disclosed were supportive and understanding:

‘The one [*teacher*] who already knows about my HIV status doesn’t ask me why I’m going to the hospital’. (C4)

#### Sub-theme 2: A need for protection against discrimination

The participants mentioned that they have a need to be protected against discrimination from their peers and teachers. Although some teachers were supportive and understanding, some were not. They often felt bullied by their peers and even by teachers, making them not want to attend school. There was no mention of previous bullying of those whose HIV status or those who are well. Participants pointed out that those students who are often a point of ridicule or made to feel uncomfortable are those who are ill or have disclosed their HIV status. One participant mentioned:

‘Some teachers are mean. They can use something you live with to be mean. I feel abused and not able to go to school’. (C6)

The participants wanted to be treated like the other children at school and not be left out of school activities because of their disease. The need for inclusion was verbalised:

‘Like when they go play sports I can play too. When they go to places like to Somhlolo [*sport stadium*], I can go too’. (C6)

### Theme 3: Experience of desire to fulfil educational needs

It came across as if the participants were dedicated to their learning and were proud of the progress they were making in school. The participants showed a desire to attend school and to learn despite their HIV-positive diagnosis.

#### Sub-theme 1: A desire to learn

The participants had a strong desire to learn and to perform in school despite the challenges they face related to their disease. One participant mentioned:

‘Yes, I’m able to learn … Sometimes I feel like my whole body is aching … Sometimes the pain then goes away, and I don’t feel it … It is something that happens very often sometimes, but I still want to learn’. (C1)

#### Sub-theme 2: Illness affecting their learning

The participants mentioned that because of their disease, they often get sick, and this influenced their school attendance. Two participants mentioned:

‘It does hurt sometimes when I am sick, it’s just that I rarely get sick … When I have a cold and headache. Then before I get better the chest becomes problematic … I can’t go to school then’. (C11)

‘Sometimes I feel pain in my heart. I normally feel this sharp pain and sometimes I cannot eat when it happens. The I can’t concentrate at school’. (C3)

#### Sub-theme 3: An expectation for teachers to be supportive in their educational needs

The participants were often absent from school, because of illness or having to access the hospital or clinic to collect their ART. There was a strong expectation among the participants for teachers to be supportive and to assist them in their educational needs when they return to school. The participants mentioned:

‘Teachers should continue to teach me, and parents should help me to study … [*when I have been absent*] I would ask the teacher to help me write what they [*the other children*] had been writing’. (C5)

‘The teacher should help me when I missed work, it’s about my well-being’. (C5)

‘The teacher must help me … I would ask the teacher to help me to catch up the work … what I have missed’. (C5)

The lived experience of the children gave an insight into the burden of going to school with HIV which only they could elucidate. Worry, sadness, loneliness and a feeling of shame and worthlessness strongly emerged from the participants.

## Discussion

The results of this study confirmed that the participants had worry and sadness about the knowledge of their HIV diagnosis. They felt alone in trying to understand and make sense of their diagnosis. Human immunodeficiency virus-positive diagnosis often creates a feeling of shame and worthlessness.^30^ It was difficult for the participants to understand HIV and its implications, but it also caused them concern to know they are labelled as HIV positive. Being HIV positive is difficult enough for an adult who understands the disease process and implications of being HIV positive, but the diagnosis is even more complex for young minds.^31^ The impact of disclosure of the HIV status of children is a topic that has not been widely researched in sub-Saharan Africa, yet in resource-rich settings, it was revealed that disclosure improves self-esteem, decreases depression and improves adherence to ART over time.^7^

Some participants felt different from other children because of being HIV positive and the long-term treatment they had to take. It made them concerned that some of their peers may view them differently. Human immunodeficiency virus-positive children often feel different from their peers.^32^ Ultimately, reassurance from their caregivers that they are not different from other children can create some sense of reassurance.^33^

Being constantly ill and missing out on school was one of the challenges participants experienced. It was difficult to cope with school demands when they felt unwell or in pain. The participants also had various specific needs because of being HIV-infected; these included the need to attend the health facility for regular check-ups and ART collection. The ART collection time was usually during school hours, and participants were thus constantly away from school, or coming in late, bringing them unwanted attention that made them uncomfortable. The participants therefore had a need to be excused for having to seek medical attention regularly without causing embarrassment or having to explain themselves. Juggling clinic appointments, getting treatment and attending school is often a challenge faced by HIV-positive children.^34^

The participants wanted to disclose to their teachers because they needed their teachers’ support, but they did not want to disclose to their peers because of the fear of discrimination by their peers. Disclosure of HIV status holds the fear of stigmatisation and discrimination, especially in children.^35,36^ Stigma and discrimination of HIV-positive children include negative attitudes and being insulted by their peers once they get to know about their positive HIV status.^36^ As a result, HIV-positive children often face verbal abuse, gossip, distancing attitudes, degradation and rejection.^36^ Those who had disclosed to teachers primarily did so because it reduced the stress of explaining their visits to the health facility for refills or when they were ill each time. Disclosure of their HIV status to their teachers is of clinical importance in children as it will assist them comply with the ART and not miss clinic visits.^37^ Some teachers would, however, ask them why they keep missing school and ask for hospital records if the children said they had been in hospital. Human immunodeficiency virus-positive children are often coerced by teachers to reveal their HIV status by requesting hospital records to prove they had been in the hospital after missing a class or school.^38^

Children’s fear of disclosing, especially to their peers, was also related to their need for protection in the school. In one study, about 45% of children felt it was important to keep their status secret, and 10% said they had lost their friends and been bullied and insulted because of their HIV status.^7^ A systematic review by Kimera et al.^39^ found that young people experienced challenges related to disclosure such as fear of gossiping, ridiculing, teasing and losing friendship. As the children concealed their status from friends, teachers and sometimes family, they also experienced depression and isolation, which was an additional challenge. It often becomes challenging for children to keep their status secret from their peers, because medication use is difficult to hide at school.^40^ Human immunodeficiency virus-related stigma affects the health and well-being of children at all stages of development, and children may often skip their ART when peers are around for fear of their HIV status being discovered.^41^

One of the participants mentioned that his teachers excluded him from certain activities that other children participated in, such as soccer, and he did not like that. This was seen as discrimination. Teachers often exempted school-going children with HIV from strenuous activities, leaving the children feeling discriminated against.^39^ To ensure a healthy transition to adulthood for children with HIV, it is critical that stigma and discrimination against children with HIV are addressed.^39^

The participants expressed that they were eager to learn despite the hardships they were facing. They knew they were left behind in some aspect of their learning and would make it a point to request assistance from teachers. Most of their difficulties in learning were because of absenteeism from school. Children with HIV should have equal access to education as well as access to treatment, including attention to special needs, physical and emotional well-being, and intellectual development.^42^ It is well known that HIV-positive children often face challenges in achieving their academic goals, and it is important that these challenges are discovered early to assist these children.^16^ The Eswatini Ministry of Education and Training^12^ confirms that it is the school’s responsibility to enhance learning and facilitate a safe and enabling environment that supports learners affected and infected with HIV. The school environment should therefore not contribute to the hardships that the school-going children with HIV are already experiencing but enhance their ease of navigating their challenges related to HIV. The Education Sector Policy in Eswatini also stipulates the need to establish schools as centres of care and support, which act as a protective, healthy, and secure learning environment that meets the interests of the ‘whole’ child.^12^

A common thread was that all the school-going children living with HIV had a strong desire to learn and perform well among their peers. Some of the participants did, however, suffer from physical symptoms, such as constant respiratory, abdominal and dermatological problems, which were deeply frustrating for them; these findings were supported by some researchers.^34,38,43^ Physical health challenges often contribute to fatigue and difficulty in concentrating at school for HIV-positive children.^43^

Schools could act as a centre-point for comprehensive support for HIV-positive children as this is the ideal place to bring teachers, parents and caregivers, peers and other support persons together to assist HIV-positive children.^44^

## Study limitations

The study included school-going children from one region in Eswatini. The other three regions in Eswatini were not included in this study. Only school-going children aged 8–13 years were included in this study. Future studies including a wider context and larger sample including adolescents may provide additional insight into the phenomenon that might not have been revealed in this study.

## Conclusions

The findings of the study suggest ways that may assist schools to support school-going children with HIV. We recommend the Eswatini Ministry of Health to design systems for educational and social support together with adequate health care for children with HIV while attending school. Both health and educational policies should involve the promotion of cooperation between health care providers, parents and caregivers, teachers and peers. We recommend that school-going children are supported and assisted by the school system to keep them in school as well as create a health promotion environment by availing therapeutic professionals and school health nurses in the school environment. Ongoing education of parents and caregivers on HIV is essential in the provision of support to HIV-positive children. The Eswatini Ministry of Health needs to continue to address the stigma around HIV and implement widespread campaigns to advocate for children with HIV in schools and the community at large. We recommend further studies to determine the reasons for HIV stigma among school-going children and teachers. Finally, we recommend a continuum of support for school-going children with HIV including the parents and caregivers, siblings, teachers, peers and other support persons.
